# Clinical Implications of Serial Glucose Measurements in Acute Ischemic Stroke Patients Treated with Intravenous Thrombolysis

**DOI:** 10.1038/s41598-018-30028-1

**Published:** 2018-08-06

**Authors:** Joon-Tae Kim, Se-Young Lee, Deok-Sang Yoo, Ji Sung Lee, Sang-Hoon Kim, Kang-Ho Choi, Man-Seok Park, Ki-Hyun Cho

**Affiliations:** 1 Department of Neurology, Chonnam National University Hospital, Gwangju, Republic of Korea; 2 Department of Neurology, KS Hospital, Gwangju, Republic of Korea; 3 Clinical Trial Center, Asan Medical Center, Seoul, Republic of Korea

## Abstract

Serial glucose might more accurately reflect glycemic status in acute ischemic stroke (AIS) than presenting glucose. We sought to investigate the clinical implications of various parameters of serial glucose on the outcomes of patients with AIS treated with intravenous thrombolysis (IVT). This was a single-center, prospective, observational study of stroke patients treated with IVT. Blood glucose (BG) was serially measured at 6-time points during the first 24 h of IVT. The primary endpoint analyzed was a good outcome at 3 m. Among the 492 patients in the cohort (age, 70 ± 12 y; men, 57%), the overall BG level was 131 ± 33 mg/dl. At 3 m, 40.4% of the patients had a good outcome. Patients with good outcomes had significantly lower mean BG (121 vs 128 mg/dl) and higher coefficient of variance (CoV, 17% vs 14%) but no differences in the others. For patients with higher mBG (every 30 mg/dl), the likelihood of achieving a good outcome decreased (OR 0.82, 95% CI 0.67–1.02). For patients with higher CoV (every 10%), the likelihood of a good outcome increased (OR 1.38, 95% CI 1.12–1.71). The results showed that higher mBG and lower CoV were consistently associated with worse outcomes in IV-thrombolyzed stroke patients, suggesting that lowering BG might be potential therapeutic target.

## Introduction

A substantial portion of patients with acute ischemic stroke (AIS) exhibit hyperglycemia at hospital admission^1,2^, and many studies have found that this hyperglycemia at admission is associated with poor clinical outcomes and increased mortality^2–4^. However, glucose levels tend to decrease over time in patients with AIS. Glucose has been shown to follow a dynamic course with an initial rise followed by a decrease and then a plateau at 14–16 h after stroke^5,6^. Therefore, a single measurement of glucose at admission might not fully reflect a patient’s glycemic status during an AIS.

The relationship between persistent hyperglycemia at 24–48 h after stroke and poor patient outcomes has been assessed, but with conflicting results^7,8^. Additionally, there is a lack of evidence indicating that intensive glucose-lowering therapy during an AIS could improve clinical outcomes^9^. Hypoglycemic episodes could potentially be neurotoxic, suggesting that serial glucose monitoring is warranted to consecutively assess the glycemic status in patients with AIS.

It is plausible that serial changes in glucose levels could be more informative than a single measurement indicating hypoglycemia or hyperglycemia at admission; however, parameters such as mean blood glucose (mBG) and glycemic variability have been consistently ignored in studies examining the association between hyperglycemia and clinical outcome in ischemic stroke^10^.

Therefore, we used serial glucose measurements to prospectively investigate the clinical implications of various glucose parameters on the outcomes in patients with AIS treated with intravenous thrombolysis (IVT).

## Methods

### Subjects

This was a single-center, prospective, observational study conducted at the Comprehensive Stroke Center of Chonnam National University Hospital between October 2012 and June 2015. Patients meeting the following inclusion criteria were consecutively enrolled: (1) AIS with ischemic lesions on apparent diffusion coefficient (ADC), (2) treatment with intravenous tissue plasminogen activator (IV-tPA) within 4.5 h of stroke onset, and (3) provision of written informed consent. Patients with any of the following criteria were excluded: (1) other etiologies based on the Trial of Org 10,172 in Acute Stroke Treatment (TOAST) classifications such as Moyamoya disease or cancer-related stroke, and (2) a premorbid modified Rankin Scale (mRS) >1. This study was approved by the Institutional Review Board of Chonnam National University Hospital and was performed in accordance with the ethical standards of the 1964 Declaration of Helsinki and its later amendments. Written informed consent was obtained from all the participants or their caregivers.

### Clinical evaluation

Demographic, clinical, and laboratory data were prospectively collected by dedicated research nurses or physicians. The following stroke risk factors were identified: hypertension, diabetes mellitus (DM), dyslipidemia, current smoking, a previous history of stroke, transient ischemic attack (TIA), or coronary artery disease. The baseline data collected from all the patients included the National Institutes of Health Stroke Scale (NIHSS) score and the stroke subtype, which was stratified according to the TOAST classification after complete diagnostic profiling with some modifications^11,12^. The NIHSS scores were assessed at admission and on each day of hospitalization by well-trained and dedicated stroke nurses.

### Blood glucose measurements

BG was measured immediately upon arrival in the Emergency Department (ED) and after IV-tPA administration using the Accu-Chek^R^ test (Accu-Chek US, Indianapolis, Indiana). Thereafter, 4 additional measurements were made, with one measurement taken every 6 h during the first 24 h after IV-tPA administration. Overall, a total of 6 measurements were acquired for the period. The following BG parameters of each patient were assessed: initial blood glucose (iBG), mBG, and maximal blood glucose (max BG). In addition, to assess glycemic variability, the standard deviation of the BG (SD), the coefficient of variance (CoV, the ratio of the SD to the mean, reported as % in the current study), and the J index were determined. The J index, which is not widely used, is derived from intermittent BG measurements and serves as a measure of both the mean level and variability of glycemia. The index is calculated as follows^13^:

$${\rm{J}}\,{\rm{index}}=0.001{({\rm{mBG}}+{\rm{SD}})}^{2}{\rm{for}}\,{\rm{glucose}}\,{\rm{measured}}\,{\rm{in}}\,{\rm{mg}}/{\rm{dl}}.$$

During hospitalization, hyperglycemia was managed using short-acting insulin in cases that exceeded 200 mg/dl according to our stroke protocol or at the physician’s discretion. Hypoglycemia was defined as ≤ 50 mg/dl of glucose and was managed using IV glucose if the patient was symptomatic or with orange juice or snack if the patient was asymptomatic.

### Outcomes

The primary clinical outcome for the study was functional independence at 3 months (a good outcome was defined as a score of 0–2 on the mRS). The secondary outcomes were the full range of disability at 3 months (all 7 levels of the mRS), symptomatic intracranial hemorrhage (SICH), which is defined as a type 2 parenchymal hemorrhage combined with neurological deterioration leading to an increase of 4 or more points on the NIHSS^14^, and death. Outcomes were assessed by dedicated stroke nurses who were blinded to glucose levels.

### Statistical analysis

The percentage, mean (±standard deviation), or median (interquartile range) is reported depending on the variable. Categorical variables were analyzed using the χ2 test and Fisher’s exact test as appropriate. Continuous variables were analyzed using the independent samples *t-*test, the Mann-Whitney U-test, analysis of variance, or the Kruskal-Wallis test as appropriate. Glucose parameters were considered continuous values, which were expressed as the change in outcome per a 30-mg/dl increase in glucose and a 10-unit increase (or 10% for CoV) in the parameters of glycemic variability. The quartiles of each parameter (each containing a quarter of the cohort) were considered categorical variables. The associations between outcomes and glucose parameters were analyzed using binary (dichotomized outcomes) or ordinal (mRS distribution) regression analysis. For ordinal analyses, each glucose parameter was dichotomized by their respective median values. Repeated measurements of BG were analyzed using the Generalized Estimating Equation (GEE). Potential confounding variables included in the adjusted analysis were predefined as age, male, baseline NIHSS score, systolic blood pressure, TOAST classification, large artery occlusion, previous stroke, endovascular therapy, atrial fibrillation, and recanalization status based on prior reports. We also analyzed the associations of the glucose parameters (continuous) with functional outcomes at 3 months according to the recanalization status after reperfusion therapy. The odds ratios (ORs) and 95% confidence intervals (CIs) were calculated. All P-values were 2-sided, and statistical significance was defined as a P-value of less than 0.05. SAS (version 9.4; SAS Institute, Cary, NC) was used for all statistical analyses. The datasets generated and analyzed during the current study are available from the corresponding author upon reasonable request.

## Results

### General characteristics

A total of 580 stroke patients treated with IVT were prospectively screened during the study period. Of these, 539 patients met the inclusion criteria for treatment with IV-tPA within 4.5 h of stroke and positive ischemic lesions based on ADC. Informed consent for the study was provided by 530 patients. Among the patients who provided informed consent, 38 were additionally excluded due to the following: 25 patients for premorbid disability (pre-mRS > 1), 4 patients for another etiology such as Moyamoya disease or cancer-related stroke, 9 patients with fewer than 3 glucose measurements, and 4 patients because of lack of mRS data at 3 months. Ultimately, 492 patients (mean age, 70 ± 12 y; men, 57.1%) were included in this study. BG was measured 6 times in 483 (98.2%) patients, 5 times in 8 patients, and 3 times in 1 patient. Of the 492 enrolled patients, 200 (40.4%) had functional independence (mRS 0–2) at 3 months, 111 (22.6%) had no or minimal disability (mRS 0–1), 22 (4.5%) had SICH, and 62 (12.5%) died.

The mean overall BG level from the serial measurements was 131 ± 33 mg/dl (median 124, IQR 110–143 mg/dl). The mean initial glucose value was 137 ± 52 mg/dl (median 125, IQR 104–152 mg/dl). The BG levels increased from the initial measurement to the third measurement, and the levels decreased with subsequent measurements (Supplemental Table S1). The maximum BG showed a similar decreasing pattern that ranged from 343 to 544 mg/dl, while the minimum BG remained relatively constant (range 54–67 mg/dl).

The general characteristics of the patients with mRS 0–2 at 3 months and SICH as well as mortality are shown in Table 1 and Supplemental Table S2. The patients who achieved independence at 3 months were younger, more frequently male, and had lower initial NIHSS scores than the patients who did not achieve this outcome (mRS 3–6). In addition, atrial fibrillation, cardioembolism on the TOAST classification, and large vessel occlusion were less frequent in the patients who achieved functional independence at 3 months.

**Table 1:** General characteristics of the study subjects according to functional outcome.

	Independent outcome (mRS 0–2)	Dependent outcome (mRS 3–6)	P-value
N	200	292	
Age, mean (SD), yr	66.3 (12.4)	72.5 (11.3)	<0.001
Male, N (%)	125 (62.5)	156 (53.4)	0.05
Initial NIHSS score (med, IQR)	8 (5, 12)	12 (9, 15.5)	<0.001
Time from onset to treatment, mean (SD), min	133 (59)	135 (59)	0.72
SBP, mean (SD), mmHg	137 (23)	139 (25)	0.23
TOAST, N (%)			0.001
LAA	45 (22.5)	64 (21.9)	
CE	54 (27.0)	125 (42.8)	
SVO	6 (3.0)	3 (1.0)	
UD	95 (47.5)	100 (34.2)	
HTN	113 (56.5)	167 (57.2)	0.93
DM	40 (20.0)	62 (21.2)	0.82
AF	20 (10.0)	54 (18.5)	0.01
Dyslipidemia	15 (7.5)	15 (5.1)	0.34
Smoking	59 (29.5)	77 (26.4)	0.47
Prior coronary disease	14 (7.0)	14 (4.8)	0.33
Prior stroke or TIA	14 (7.0)	34 (11.6)	0.09
Large artery occlusion	119 (59.5)	226 (77.4)	<0.001
MCA	25 (12.5)	34 (11.6)	
Intracranial ICA	47 (23.5)	83 (28.4)	
Extracranial ICA	35 (17.5)	84 (28.8)	
Vertebrobasilar	3 (1.5)	14 (4.8)	
Others	9 (4.5)	11 (3.8)	
Endovascular therapy	58 (29.0)	73 (25.0)	0.35
Insulin therapy	13 (6.5)	26 (8.9)	0.40
Glucose parameters, median (IQR), mg/dl			
Mean blood glucose	120.7 (106.5, 137.5)	127.5 (112.5, 146)	0.002
Initial blood glucose	124 (101, 151)	126 (107, 153)	0.31
Maximal blood glucose	152 (128, 188)	156.5 (131.5, 181)	0.43
Glycemic variability, median (IQR)			
Standard deviation, unit	21 (12.5, 32.2)	18 (12.5, 27)	0.13
Coefficient of variance, %	17 (11.2, 23.5)	14.3 (10.2, 19.8)	0.005
J index, unit	20.2 (15.1, 27.3)	21.7 (16.3, 28.8)	0.11

Abbreviations: NIHSS, National Institutes of Health Stroke Scale; SBP, systolic blood pressure; TOAST, Trials of Org 10,172 in Acute Stroke Treatment; LAA, large artery atherosclerosis; CE, cardioembolism; SVO, small vessel occlusion; UD, undetermined etiology; HTN, hypertension; DM, diabetes mellitus; AF, atrial fibrillation; TIA, transient ischemic attack; MCA, middle cerebral artery; ICA, internal carotid artery.

### Glucose parameters and outcomes

The patients with functional independence at 3 months had significantly lower mBG levels (127 vs. 134 mg/dl, P = 0.01) than patients who did not achieve independence, but no significant differences were observed in iBG or max BG values. In terms of glycemic variability, the CoV was significantly higher in the patients with independent outcomes than in patients with dependent outcomes, but the SD and J index did not differ (Table 1). Comparisons of clinical outcomes based on quartiles with glucose as the categorical variable are shown in Table 2 and Supplemental Table S3. The proportions of patients in the various mBG and CoV quartiles were significantly different between patients who did and did not achieve an independent outcome at 3 months (Table 2). Specifically, a greater proportion of patients with functional independence was found in the lowest quartiles for mBG than in the highest quartiles. By contrast, a greater proportion of independent patients were in the highest quartiles of CoV than in the lowest quartiles.

**Table 2:** Categorical glucose parameters and functional outcomes.

	Independent outcome (mRS 0–2)	Dependent outcome (mRS 3–6)	P-value
N	200	292	
Initial blood glucose, n (%)			0.50
1Q (≤104 mg/dl)	58 (29.0)	67 (22.9)	
2Q (105–125 mg/dl)	48 (24.0)	77 (26.4)	
3Q (126–152 mg/dl)	49 (24.5)	74 (25.3)	
4Q (≥153 mg/dl)	45 (22.5)	74 (25.3)	
Mean blood glucose, n (%)			0.005
1Q (≤110 mg/dl)	64 (32.0)	61 (20.9)	
2Q (111–124 mg/dl)	54 (27.0)	68 (23.3)	
3Q (125–143 mg/dl)	45 (22.5)	78 (26.7)	
4Q (≥144 mg/dl)	37 (18.5)	85 (29.1)	
Maximum blood glucose, n (%)			0.12
1Q (≤131 mg/dl)	54 (27.0)	69 (23.6)	
2Q (132–154 mg/dl)	55 (27.5)	68 (23.3)	
3Q (155–182 mg/dl)	39 (19.5)	85 (29.1)	
4Q (≥183 mg/dl)	52 (26.0)	70 (24.0)	
Standard deviation, n (%)			0.28
1Q (<12.5)	48 (24.0)	76 (26.0)	
2Q (12.5–18.9)	46 (23.0)	77 (26.4)	
3Q (18.9–28.3)	46 (23.0)	74 (25.3)	
4Q (≥28.4)	60 (30.0)	65 (22.3)	
Coefficient of variance, n (%)			0.005
1Q (≤0.1)	48 (24.0)	80 (27.4)	
2Q (0.1–0.15)	39 (19.5)	86 (29.5)	
3Q (0.15–0.21)	54 (27.0)	75 (25.7)	
4Q (>0.21)	59 (29.5)	51 (17.5)	
J index, n (%)			0.42
1Q (≤15.8)	57 (28.5)	66 (22.6)	
2Q (15.8–21.0)	51 (25.5)	72 (24.7)	
3Q (21.0–28.4)	45 (22.5)	78 (26.7)	
4Q (>28.4)	47 (23.5)	76 (26.0)	

The quartiles of each parameter contained a quarter of the cohort population.

Table 3 and Supplemental Table S4 provide unadjusted and adjusted ORs for the binary outcomes with glucose as both a continuous and categorical variable. Increased mBG levels were associated with a decreased likelihood of achieving functional independence at 3 months as well as with increased mortality. For every 30-mg/dl increase in mBG, a good outcome (mRS 0–2) at 3 months was less likely to occur (OR 0.82, 95% CI 0.67–1.01), but SICH (OR 1.51, 95% CI 1.11–2.06) and mortality (OR 1.26, 95% CI 0.98–1.60) were more likely to occur. For every 10% increase in CoV, an independent outcome at 3 months was more likely to occur (OR 1.38, 95% CI 1.12–1.71). A similar but nonsignificant association was observed between SD and independent outcome (OR 1.11, 95% CI 0.99–1.25). Other parameters, including the iBG, the max BG, and the J index, were not associated with an independent outcome at 3 months, SICH, or mortality. Compared with the patients in the first quartile of mBG (≤110 mg/dl), patients in the fourth quartile (≥144 mg/dl) were less likely to have an independent outcome at 3 months (OR 0.44, 95% CI 0.24–0.81) and more likely to experience SICH (OR 9.27, 95% CI 1.75–49.3) and death (OR 3.13, 95% CI 1.11–8.61). Compared with the patients in the first quartile of CoV, patients in the fourth quartile were more likely to achieve an independent outcome at 3 months (OR 1.91, 95% CI 1.07–3.43), but there was no difference in SICH or mortality risk. Regarding the assessed parameters of glycemic variability, especially the SD and J index, the patients in the higher quartiles of glucose variability were more likely to have an increased risk of mortality than patients in the first quartile.

**Table 3:** Associations between various glucose parameters and clinical outcomes.

	mRS 0–2 at 3 months			
	Unadjusted OR (95% CI)	P-value	Adjusted OR (95% CI)	P-value
Initial BG				
for every 30-mg/dl increase	0.98 (0.88–1.09)	0.72	1.01 (0.90–1.14)	0.87
1Q	Ref		Ref	
2Q	0.72 (0.44–1.19)	0.20	0.80 (0.45–1.42)	0.44
3Q	0.76 (0.46–1.27)	0.30	0.77 (0.44–1.38)	0.38
4Q	0.70 (0.42–1.17)	0.18	0.75 (0.42–1.33)	0.33
Mean BG				
for every 30-mg/dl increase	0.79 (0.66–0.95)	0.01	0.82 (0.67–1.01)	0.06
1Q	Ref		Ref	
2Q	0.76 (0.46–1.25)	0.28	0.81 (0.46–1.44)	0.48
3Q	0.55 (0.33–0.91)	0.02	0.57 (0.32–1.01)	0.05
4Q	0.41 (0.25–0.70)	<0.001	0.44 (0.24–0.81)	0.01
Max BG				
for every 30-mg/dl increase	1.00 (0.91–1.10)	0.95	1.01 (0.91–1.13)	0.85
1Q	Ref		Ref	
2Q	1.03 (0.62–1.71)	0.90	1.30 (0.73–2.31)	0.37
3Q	0.59 (0.35–0.99)	0.04	0.62 (0.34–1.11)	0.10
4Q	0.95 (0.57–1.57)	0.84	0.99 (0.56–1.77)	0.98
SD				
for every 10-unit increase	1.11 (1.00–1.23)	0.04	1.11 (0.99–1.25)	0.08
1Q	Ref		Ref	
2Q	0.95 (0.57–1.58)	0.83	1.04 (0.58–1.86)	0.89
3Q	0.98 (0.59–1.65)	0.95	1.08 (0.61–1.92)	0.80
4Q	1.46 (0.88–2.42)	0.14	1.40 (0.80–2.47)	0.24
CoV				
for every 10% increase	1.40 (1.16–1.68)	<0.001	1.38 (1.12–1.71)	0.003
1Q	Ref		Ref	
2Q	0.76 (0.45–1.27)	0.29	0. 802 (0.44–1.43)	0.49
3Q	1.20 (0.73–1.98)	0.48	1.27 (0.73–2.23)	0.40
4Q	1.93 (1.15–3.24)	0.01	1.91 (1.07–3.43)	0.03
J index				
for every 10-unit increase	0.96 (0.88–1.06)	0.45	0.97 (0.88–1.08)	0.63
1Q	Ref		Ref	
2Q	0.82 (0.50–1.36)	0.44	0.83 (0.47–1.47)	0.52
3Q	0.67 (0.40–1.11)	0.12	0.75 (0.42–1.35)	0.34
4Q	0.72 (0.43–1.19)	0.20	0.76 (0.42–1.37)	0.36

BGs, OR for every 30-mg/dl increase in BG; SD and J index, OR for every 10-unit increase; CoV, OR for every 10% increase.

Adjusted variables: age, male, initial NIHSS score, TOAST classification, endovascular therapy, AF, prior stroke, large artery occlusion, SBP, and recanalization status.

**Figure 1:**
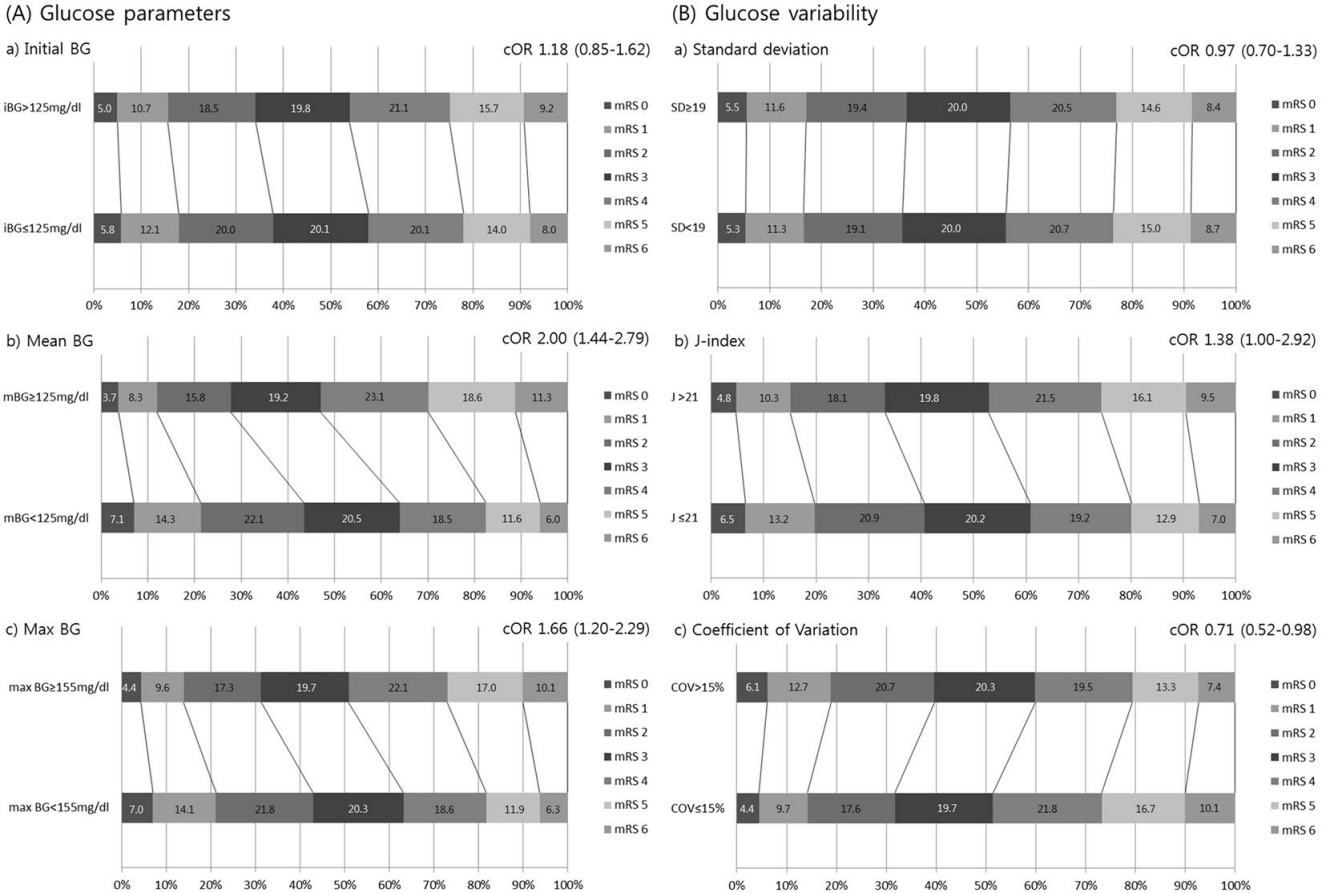
Adjusted distributions across the entire modified Rankin Scale (mRS) at 3 months in patients according to the dichotomous parameters of glucose (**A**) and glycemic variability (**B**).

The functional outcome at 3 months across the entire ordinal range of mRS was independently linked to dichotomous mBG (≥125 vs. <125 mg/dl, based on the median mBG value) and max BG (≥155 vs. <155 mg/dl, based on the median max BG value). For higher mBG and max BG, worse functional outcomes at 3 months were more likely to occur (mBG: cOR 2.00, 95% CI 1.44–2.79; max BG: cOR 1.66, 95% CI 1.20–2.29) (Fig. 1). Likewise, for higher J index (>21) values, worse functional outcomes at 3 months were more likely to occur (cOR 1.38, 95% CI 1.00–1.92). By contrast, for higher CoVs (>15%), better functional outcomes at 3 months were more likely to occur (cOR 0.71, 95% CI 0.52–0.98).

The relationship between glucose parameters and clinical outcomes stratified for recanalization status at follow-up angiography is shown in Table 4. While among patients with complete recanalization, for every 30-mg/dl increase in mBG, a good outcome (mRS 0–2) at 3 months was less likely to occur (OR 0.77, 95% CI 0.61–0.98), and no association of mBG with a good outcome at 3 months was observed among patients with incomplete recanalization (OR 0.95, 95% CI 0.82–1.09). Among patients with incomplete recanalization, for every 10% increase in CoV, an independent outcome at 3 months was likely to occur (OR 2.35, 95% CI 1.42–3.87) but not among patients with complete recanalization.

**Table 4:** Association between glucose parameters and functional outcomes stratified according to recanalization status.

	Independent outcome (mRS 0–2)	Dependent outcome (mRS 3–6)	P-value	Adjusted OR (95% CI)	P-value
Complete recanalization (N = 348)	N = 167	N = 181			
Initial BG	135.5 (53.0)	137.4 (47.4)	0.72	0.99 (0.87–1.14)	0.94
Mean BG	126.8 (30.5)	134.5 (32.7)	0.02	0.77 (0.61–0.98)	0.03
Max BG	167.1 (64.5)	167.6 (48.9)	0.94	0.98 (0.86–1.10)	0.71
SD	25.3 (21.6)	22.4 (14.3)	0.15	1.06 (0.93–1.21)	0.39
CoV	18.8 (12.3)	16.3 (8.3)	0.02	1.20 (0.95–1.52)	0.13
J index	25.3 (19.6)	26.4 (18.3)	0.59	0.96 (0.85–1.08)	0.48
Incomplete recanalization (N = 144)	N = 33	N = 111			
Initial BG	136.5 (48.6)	137.3 (58.1)	0.94	1.00 (0.93–1.09)	0.92
Mean BG	125.9 (28.8)	133.8 (37.4)	0.27	0.95 (0.82–1.09)	0.45
Max BG	168.0 (39.2)	165.8 (59.4)	0.84	1.02 (0.94–1.10)	0.63
SD	27.5 (13.6)	22.0 (17.4)	0.10	1.25 (0.97–1.60)	0.08
CoV (%)	22.3 (11.0)	15.6 (8.3)	<0.001	2.35 (1.42–3.87)	0.001
J index	24.8 (12.6)	26.9 (23.3)	0.62	0.97 (0.77–1.22)	0.09

Adjusted variables: age, male, initial NIHSS score, TOAST classification, endovascular therapy, AF, prior stroke, large artery occlusion, and SBP.

### Serial glucose measurements and outcomes

**Figure 2:**
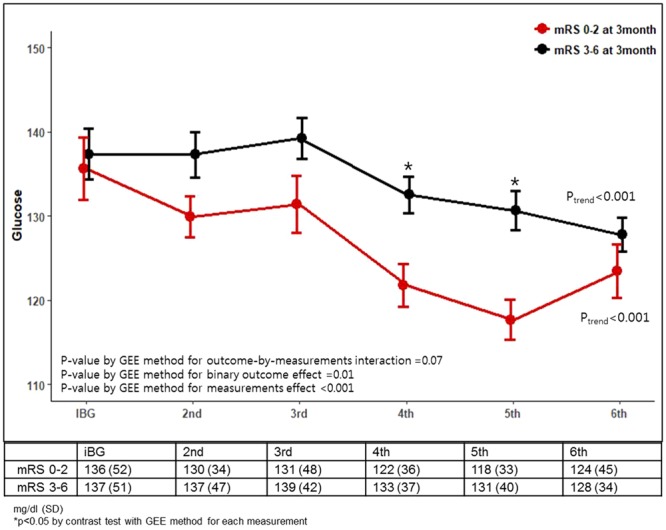
Serial glucose levels plotted according to the 3-month independent vs. dependent outcomes.

Figure 2 and Supplemental Fig. S1 shows the relationship among changes in serial glucose levels, functional outcomes, SICH, and mortality. A weak but nonsignificant association was observed between serial measurements and functional outcome (independent vs. dependent) (P_interaction_ = 0.07 by GEE methods for mRS-by-measurement interaction). Patterns of decreasing serial BG levels were observed in patients with both independent and dependent outcomes (both P_trend_ < 0.001), but the patients who achieved functional independence had significantly lower serial BG values than patients who did not achieve this outcome (P = 0.02 by GEE method for mRS effect). An interaction was observed between serial glucose levels and both SICH (P_interaction_ = 0.02 by GEE methods for SICH-by-measurement interaction) and mortality (P_interaction_ = 0.03 by GEE methods for mortality-by-measurement interaction). BG levels substantially decreased over time in the patients without either SICH or mortality (both P_trend_ < 0.001) but not in those with SICH or mortality (P_trend_ = 0.26 and 0.68, respectively).

## Discussion

In the current prospective study of 492 AIS patients treated with IVT, we examined various glucose parameters during the first 24 h after IVT. The results showed that higher mBG was most consistently associated with an increased risk of worse clinical outcomes, including dependent functional outcome, SICH and mortality, whereas other parameters, including iBG and max BG, seemed to be either weak predictors or not predictors of these outcomes. Regarding the glycemic variability, a good outcome at 3 months was more likely to occur in patients with a higher CoV. In addition, patients with better outcomes showed greater decreases in serial glucose values during the first 24 h after IVT. Therefore, these results suggest that compared with a single glucose measurement, serial glucose measurements could be more informative in predicting functional outcomes of AIS patients treated with IVT.

In our study, for every increase of 30 mg/dl in mBG, a 19% reduction of achieving a good outcome at 3 months, a 49% increase in the risk of SICH, and a 26% increase in the risk of mortality were observed. These results were consistent with the results of a previous study, which showed that a mBG value over 180 mg/dl was predictive of worse outcomes^10^. In addition, persistent hyperglycemia was reported as an independent determinant of infarct expansion and was thus associated with worse clinical outcomes^15^; specifically, mean glucose levels ≥125 mg/dl over the monitoring period correlated with MRI outcomes.

The results of our study suggested the detrimental effects of high glucose levels on stroke outcomes. Several pathophysiological mechanisms have been suggested to account for the detrimental effect of hyperglycemia observed in patients with AIS. In a previous study, transcranial Doppler imaging showed that hyperglycemia was linked with persistent occlusion after tPA treatment^16^. Additionally, hyperglycemia or insulin resistance could impair recanalization through increased coagulation and decrease fibrinolytic activity^17–19^. Furthermore, decreased reperfusion to the ischemic tissue and increased infarct volumes were related with hyperglycemia^20,21^. Inhibition of vasodilatation is an important mechanism by which hyperglycemia seems to reduce cerebral blood flow^22,23^. Moreover, glycemic control before stroke occurrence was an independent prognostic factor in patients with ischemic stroke, and HbA1c values above the recommended goals increased the risk of an unfavorable 3-month outcome by the impairment of neurological plasticity and vascular recovery^24^. Both acute and chronic hyperglycemia are associated with increased mortality and worse clinical outcomes in AIS patients treated with tPA.

Therefore, our findings provide supportive evidence that modest-to-intensive glucose lowering, especially with regard to achieving a mBG < 125 mg/dl, might improve functional outcomes in AIS. mBG values ≥ 125 mg/dl (vs. < 125 mg/dl) were independently associated with a 71% greater risk of an increased degree of disability at 3 months. Although there is no evidence that insulin therapy benefits patients with AIS^9^, aggressive glucose-reduction treatment via the delivery of continuous intravenous insulin in patients with baseline glucose values ≥ 150 mg/dl was associated with nonsignificant improvements in outcomes in a pilot trial^25^. Given that the benefit of glucose-lowering treatment could be negated by hypoglycemic episodes^26,27^, modest-to-intensive glucose lowering to achieve reductions in the range of 80–125 mg/dl without symptomatic hypoglycemia might potentially benefit patients with AIS after they undergo IVT. However, because our study was not designed to evaluate the effects of any potential therapy, the results of our study should be interpreted with caution and further studies are needed to confirm our results. Ongoing research (NCT01369069) is expected to provide more consistent answers for intensive vs. standard glucose management in AIS.

Glycemic variability has been identified as a predictor of hypoglycemia and has been related to intensive care unit mortality^28,29^. One prior study found that the incidence of cerebral infarction was 64% for patients with spontaneous subarachnoid hemorrhage who had high glycemic variability^30^. Unlike prior studies reporting that increased glycemic variability was more likely to result in worse outcomes^31–33^, in the current study, higher CoV values were more likely to have a good 3-month outcome by 37% for every 10% increase. Consistently, less than 15% of CoV was associated with worse functional outcomes at 3 months (cOR 0.71). The impact of higher CoV on a good outcome was more substantial in patients with incomplete recanalization, but similar trends were also observed in patients with complete recanalization. Our study differed from previous studies because no symptomatic hypoglycemia was observed; thus, the impact of a higher CoV on outcome may be unusual. Higher values of CoV in the current study represent greater extent of decreasing serial BG levels, likely having a favorable outcome as shown in Fig. 2.

In addition, our study showed that different parameters of glycemic variability have different implications on clinical outcomes. In contrast to the CoV, the J index values in the highest quartile were associated with increased mortality and SICH compared with values in the lowest quartile. Considering that different equations were applied for each parameter of glycemic variability, the SD and the CoV were well correlated with the changes in the mean BG, whereas the J index appeared to be more representative of the values of the mean BG; therefore, it is plausible that these parameters have different clinical implications. Further studies are thus needed to better understand the clinical implications of different parameters associated with glycemic variability.

The dynamics of the glucose levels during the first 24 h after IVT differed according to clinical outcomes. Patients with worse outcomes, especially mortality and SICH, exhibited fewer patterns of decreased glucose values after IVT than those with better outcomes. Previous studies found that persistent hyperglycemia at 24 h was associated with worse outcomes, but these interpretations are limited because either only 2 measurements of glucose values were available or there was heterogeneity among studies regarding the definition of persistent hyperglycemia^8,34^. Our study investigated the dynamics of BG more accurately; thus, these results support the assumption for implementing modest-to-intensive glucose-lowering therapy during the immediate periods after AIS.

Previous studies showed that the impact of hyperglycemia on outcome could be different in patients according to the recanalization or reperfusion status^4,35^. Our results are consistent with the results of a prior study in which the admission hyperglycemia was more likely to have a poor outcome in reperfused tPA-treated patients but not in nonreperfused tPA-treated patients^4^. By contrast, in a post hoc study of mechanical thrombectomy, the magnitude of the negative impact of hyperglycemia on the functional outcome was more substantial in patients with incomplete recanalization^35^. Given that hyperglycemia acts on the tPA response but not in mechanical thrombectomy, the impact of recanalization on glycemic status might be different according to the treatment. Further study is thus warranted.

A variety of factors related to metabolic homeostasis, inflammatory response, cerebral perfusion disturbances, and pharmacological actions could have an influence on stroke outcome. All these factors may act at either the brain site of damage or at the systemic level, influencing the neurovascular recovery, secondary damage, and systemic complications. Accordingly, more understanding of stroke pathophysiology can identify the biochemical, clinical or imaging parameters that may contribute to improve the stroke outcome could^24,36–40^. Furthermore, as many of these variables can be controlled, they could even become future targets of treatment in stroke patients. Therefore, a multidimensional evaluation would be important for stroke management.

This study had several limitations. First, we measured BG only during the first 24 h after IVT; therefore, the impact of BG levels on clinical outcome beyond 24 h could not be assessed. However, BG typically decreases and plateaus at 14–16 h after stroke, and studying the 24-h window after IVT might be acceptable. Second, this observational study only documented associations and did not investigate causative relationships. Third, we did not investigate the effect of insulin treatment on patients with hyperglycemia. In addition, the absolute differences in mBG levels between independent and dependent outcomes at 3 months were only 7 mg/dl, which does not appear to be clinically significant. Future studies should investigate appropriate methods and targets for glucose management. Additionally, we did not provide data on blood pressure (BP) or BP variability, but high BP and BP variations are important predictors of stroke prognosis^41,42^. Similarly, we did not provide data regarding the patients’ medications at baseline, despite the effects of many different drugs on both metabolic indices and BP control^43^.

## Conclusions

In conclusion, among the various glucose parameters assessed in patients with AIS, we found that a higher mean BG was consistently associated with worse clinical outcomes. By contrast, a higher CoV was more likely to have good outcome. Our results suggest that serial BG monitoring could be helpful in predicting outcomes in patients with AIS and that the lowering of higher BG without episodic hypoglycemia might be a potential target for glucose management in AIS patients.

## Electronic supplementary material

Supplementary Information
