# Supplementary material for: Clinical Implications of Serial Glucose Measurements in Acute Ischemic Stroke Patients Treated with Intravenous Thrombolysis
Source: Sci Rep. 2018 Aug 6;8:11761. doi: 10.1038/s41598-018-30028-1 (PMC6078974; doi:10.1038/s41598-018-30028-1)

**Supplemental Materials**

**Title:** Clinical Implications of Serial Glucose Measurements in Acute Ischemic Stroke Patients Treated with Intravenous Thrombolysis

Tables: 4

Figures: 1

Supplemental Table S1. Changes in serial glucose measurements in patients with acute ischemic stroke

|  | iBG | 2nd | 3rd | 4th | 5th | 6th |
| --- | --- | --- | --- | --- | --- | --- |
| N | 492 | 492 | 492 | 491 | 491 | 483 |
| Mean BG (SD) | 137 (52) | 134 (42) | 136 (45) | 128 (37) | 125 (38) | 126 (39) |
| Median BG (IQR) | 125 (48) | 126 (43) | 126 (46) | 120 (41) | 117 (39) | 117 (40) |
| Maximum BG | 486 | 441 | 544 | 384 | 343 | 368 |
| Minimum BG | 54 | 65 | 67 | 60 | 63 | 58 |

Abbreviations: iBG, initial blood glucose; BG, blood glucose.

Supplemental Table S2. General characteristics of the study subjects

|  | SICH | No SICH | P-value | Death | Alive | P-value |
| --- | --- | --- | --- | --- | --- | --- |
| N | 22 | 470 |  | 62 | 430 |  |
| Age, mean (SD), yr | 69.9 (16.5) | 70.0 (11.9) | 0.34 | 74.8 (8.8) | 69.2 (12.4) | <0.001 |
| Male, N (%) | 9 (40.9) | 272 (57.9) | 0.13 | 33 (53.2) | 178 (41.4) | 0.10 |
| Initial NIHSS (med, IQR) | 12.5 (10, 15) | 10 (7, 14) | 0.10 | 14.5 (12, 18) | 10 (6, 14) | <0.001 |
| Time from onset to treatment,  mean (SD), min | 144.2 (65.9) | 134.2 (58.4) | 0.59 | 133.6 (58.3) | 134.8 (58.8) | 0.89 |
| SBP, mean (SD), mmHg | 135.5 (28) | 138.3 (24) | 0.30 | 135 (26) | 139 (24) | 0.24 |
| TOAST, N (%) |  |  | 0.17 |  |  | 0.002 |
| LAA | 1 (4.5) | 108 (23.0) |  | 5 (8.1) | 104 (24.2) |  |
| CE | 11 (50.0) | 168 (35.7) |  | 34 (54.8) | 145 (33.7) |  |
| SVO | 0 | 9 (1.9) |  | 0 | 9 (2.1) |  |
| UD | 10 (45.5) | 185 (39.4) |  | 23 (37.1) | 172 (40.0) |  |
| HTN | 13 (59.1) | 267 (56.8) | >0.99 | 35 (56.5) | 245 (57.0) | >0.99 |
| DM | 4 (18.2) | 98 (20.9) | >0.99 | 14 (22.6) | 88 (20.5) | 0.74 |
| AF | 5 (22.7) | 69 (14.7) | 0.35 | 14 (22.6) | 60 (14.0) | 0.09 |
| Dyslipidemia | 1 (4.5) | 29 (6.2) | >0.99 | 2 (3.2) | 28 (6.5) | 0.41 |
| Smoking | 4 (18.2) | 132 (28.1) | 0.46 | 15 (24.2) | 121 (28.1) | 0.65 |
| Prior coronary disease | 1 (4.5) | 27 (5.7) | >0.99 | 5 (8.1) | 23 (5.3) | 0.38 |
| Prior stroke or TIA | 3 (13.6) | 45 (9.6) | 0.46 | 7 (11.3) | 41 (9.5) | 0.65 |
| Large artery occlusion | 17 (77.3) | 328 (69.8) | 0.63 | 51 (82.3) | 294 (68.4) | 0.03 |
| MCA | 3 (13.6) | 56 (11.9) |  | 6 (9.7) | 53 (12.3) |  |
| Intracranial ICA | 7 (31.8) | 123 (26.2) |  | 15 (24.2) | 115 (26.7) |  |
| Extracranial ICA | 5 (22.7) | 114 (24.3) |  | 24 (38.7) | 95 (22.1) |  |
| Vertebrobasilar | 0 | 17 (3.6) |  | 4 (6.5) | 13 (3.0) |  |
| Others | 2 (9.1) | 18 (3.8) |  | 2 (3.2) | 18 (4.2) |  |
| Endovascular therapy | 4 (18.2) | 127 (27.0) | 0.46 | 12 (19.4) | 119 (27.7) | 0.22 |
| Insulin sliding | 4 (18.2) | 35 (7.4) | 0.09 | 5 (8.1) | 34 (7.9) | >0.99 |
| Glucose parameters,  mean (SD), mg/dl |  |  |  |  |  |  |
| Mean blood glucose | 149 (31) | 130 (33) | 0.01 | 139 (28) | 130 (33) | 0.001 |
| Initial blood glucose | 141 (37) | 136 (52) | 0.57 | 139 (44) | 136 (53) | 0.27 |
| Maximal blood glucose | 183 (46) | 166 (57) | 0.18 | 171 (42) | 166 (58) | 0.06 |
| Glycemic variability,  mean (SD) |  |  |  |  |  |  |
| Standard deviation, unit | 25 (15) | 24 (18) | 0.79 | 23 (12) | 24 (19) | 0.27 |
| Coefficient of variance, % | 16 (8) | 17 (10) | 0.47 | 16 (7) | 18 (11) | 0.27 |
| J index, unit | 32 (16) | 26 (20) | 0.15 | 28 (13) | 26 (20) | 0.009 |

Supplemental Table S3. Categorical glucose parameters and clinical outcomes

|  | SICH | No SICH | P-value | Death | Alive | P-value |
| --- | --- | --- | --- | --- | --- | --- |
| N | 22 | 470 |  | 62 | 430 |  |
| Initial blood glucose, n (%) |  |  | 0.24 |  |  | 0.21 |
| 1Q (≤104) | 2 (9.1) | 123 (26.2) |  | 13 (21.0) | 112 (26.0) |  |
| 2Q (105-125) | 7 (31.8) | 118 (25.1) |  | 15 (24.2) | 110 (25.6) |  |
| 3Q (126-152) | 5 (22.7) | 118 (25.1) |  | 15 (24.2) | 108 (25.1) |  |
| 4Q (≥153) | 8 (36.4) | 111 (23.6) |  | 19 (30.6) | 100 (23.3) |  |
| Mean blood glucose |  |  | 0.002 |  |  | 0.001 |
| 1Q (≤ 110) | 2 (9.1) | 123 (26.2) |  | 7 (11.3) | 118 (27.4) |  |
| 2Q (111-124) | 1 (4.5) | 121 (25.7) |  | 12 (19.4) | 110 (25.6) |  |
| 3Q (125-143) | 7 (31.8) | 116 (24.7) |  | 22 (35.5) | 101 (23.5) |  |
| 4Q (≥144) | 12 (54.5). | 110 (23.4) |  | 21 (33.9) | 101 (23.5) |  |
| Maximum blood glucose |  |  | 0.05 |  |  | 0.17 |
| 1Q (≤131) | 2 (9.1) | 121 (25.7) |  | 10 (16.1) | 113 (26.3) |  |
| 2Q (132-154) | 3 (13.6) | 120 (25.5) |  | 13 (21.0) | 110 (25.6) |  |
| 3Q (155-182) | 10 (45.5) | 114 (24.3) |  | 20 (32.3) | 104 (24.2) |  |
| 4Q (≥183) | 7 (31.8) | 115 (24.5) |  | 19 (30.6) | 103 (24.0) |  |
| Standard deviation |  |  | 0.08 |  |  | 0.06 |
| 1Q (<12.5) | 5 (22.7) | 119 (25.3) |  | 8 (12.9) | 116 (27.0) |  |
| 2Q (12.5-18.9) | 2 (9.1) | 121 (25.7) |  | 22 (35.5) | 101 (23.5) |  |
| 3Q (18.9-28.3) | 10 (45.5) | 110 (23.4) |  | 15 (24.2) | 105 (24.4) |  |
| 4Q (≥28.4) | 5 (22.7) | 120 (25.5) |  | 17 (27.4) | 108 (25.1) |  |
| Coefficient of variance |  |  | 0.48 |  |  | 0.64 |
| 1Q (≤0.1) | 6 (27.3) | 122 (26.0) |  | 11 (17.7) | 117 (27.2) |  |
| 2Q (0.1-0.15) | 7 (31.8) | 118 (25.1) |  | 24 (38.7) | 101 (23.5) |  |
| 3Q (0.15-0.21) | 7 (31.8) | 122 (26.0) |  | 19 (30.6) | 110 (25.6) |  |
| 4Q (>0.21) | 2 (9.1) | 108 (23.0) |  | 8 (12.9) | 102 (23.7) |  |
| J index |  |  | 0.02 |  |  | 0.002 |
| 1Q (≤15.8) | 2 (9.1) | 121 (25.7) |  | 7 (11.3) | 116 (27.0) |  |
| 2Q (15.8-21.0) | 2 (9.1) | 121 (25.7) |  | 13 (21.0) | 110 (25.6) |  |
| 3Q (21.0-28.4) | 10 (45.5) | 113 (24.0) |  | 21 (33.9) | 102 (23.7) |  |
| 4Q (>28.4) | 8 (36.4) | 115 (24.5) |  | 21 (33.9) | 102 (23.7) |  |

Supplemental Table S4. Associations between various glucose parameters and clinical outcomes

|  | SICH | | | | Mortality | | | |
| --- | --- | --- | --- | --- | --- | --- | --- | --- |
|  | Unadjusted | P-value | Adjusted | P-value | Unadjusted | P-value | Adjusted | P-value |
| Initial BG | 1.02 (0.94-1.10) | 0.67 | 1.05 (0.82-1.35) | 0.69 | 1.03 (0.89-1.19) | 0.68 | 1.01 (0.85-1.20) | 0.91 |
| 1Q | Ref |  | Ref |  | Ref |  | Ref |  |
| 2Q | 3.65 (0.74-17.92) | 0.11 | 4.33 (0.85-22.1) | 0.08 | 1.17 (0.53-2.58) | 0.69 | 1.01 (0.42-2.44) | 0.98 |
| 3Q | 2.61 (0.50-13.69) | 0.26 | 2.70 (0.49-14.9) | 0.25 | 1.20 (0.54-2.63) | 0.66 | 0.99 (0.41-2.42) | 0.98 |
| 4Q | 4.43 (0.92-21.32) | 0.06 | 4.47 (0.89-22.8) | 0.07 | 1.64 (0.77-3.48) | 0.20 | 1.43 (0.61-3.36) | 0.41 |
| Mean BG | 1.12 (1.03-1.23) | 0.01 | 1.51 (1.11-2.06) | 0.01 | 1.24 (1.01-1.23) | 0.04 | 1.26 (0.98-1.60) | 0.07 |
| 1Q | Ref |  | Ref |  | Ref |  | Ref |  |
| 2Q | 0.51 (0.05-5.68) | 0.59 | 0.62 (0.05-7.26) | 0.71 | 1.84 (0.70-4.84) | 0.22 | 1.88 (0.65-5.36) | 0.24 |
| 3Q | 3.71 (0.76-18.23) | 0.11 | 4.03 (0.78-20.9) | 0.10 | 3.67 (1.51-8.95) | 0.004 | 4.07 (1.53-10.9) | 0.01 |
| 4Q | 6.71 (1.47-30.64) | 0.01 | 9.27 (1.75-49.3) | 0.01 | 3.50 (1.43-8.58) | 0.006 | 3.13 (1.11-8.61) | 0.03 |
| Max BG | 1.04 (0.98-1.11) | 0.18 | 1.15 (0.95-1.40) | 0.15 | 1.05 (0.92-1.20) | 0.48 | 1.07 (0.91-1.25) | 0.43 |
| 1Q | Ref |  | Ref |  | Ref |  | Ref |  |
| 2Q | 1.51 (0.25-9.21) | 0.65 | 1.24 (0.20-7.72) | 0.82 | 1.34 (0.56-3.17) | 0.51 | 1.07 (0.41-2.76) | 0.89 |
| 3Q | 5.31 (1.14-24.75) | 0.03 | 5.11 (1.05-24.9) | 0.04 | 2.17 (0.97-4.86) | 0.06 | 2.50 (1.01-6.22) | 0.05 |
| 4Q | 3.68 (0.75-18.10) | 0.11 | 3.67 (0.70-19.2) | 0.12 | 2.08 (0.93-4.69) | 0.08 | 2.21 (0.88-5.61) | 0.09 |
| SD | 1.03 (0.82-1.29) | 0.79 | 1.04 (0.81-1.33) | 0.75 | 0.97 (0.83-1.14) | 0.74 | 1.00 (0.84-1.21) | 0.98 |
| 1Q | Ref |  | Ref |  | Ref |  | Ref |  |
| 2Q | 0.39 (0.07-2.07) | 0.27 | 0.37 (0.07-2.01) | 0.25 | 3.16 (1.35-7.40) | 0.01 | 2.94 (1.17-7.40) | 0.02 |
| 3Q | 2.16 (0.72-6.53) | 0.17 | 2.13 (0.67-6.73) | 0.20 | 2.07 (0.84-5.08) | 0.11 | 2.14 (0.81-5.63) | 0.12 |
| 4Q | 0.99 (0.28-3.51) | 0.99 | 0.94 (0.25-3.51) | 0.92 | 2.28 (0.95-5.50) | 0.07 | 2.74 (1.10-7.19) | 0.04 |
| CoV | 0.84 (0.52-137) | 0.50 | 0.83 (0.49-1.40) | 0.47 | 0.86 (0.64-1.15) | 0.31 | 0.92 (0.65-1.30) | 0.64 |
| 1Q | Ref |  | Ref |  | Ref |  | Ref |  |
| 2Q | 1.21 (0.39-3.69) | 0.74 | 1.24 (0.39-3.97) | 0.72 | 2.53 (1.18-5.41) | 0.02 | 2.62 (1.12-6.12) | 0.03 |
| 3Q | 1.17 (0.38-3.57) | 0.79 | 1.12 (0.35-3.57) | 0.85 | 1.84 (0.84-4.04) | 0.13 | 2.10 (0.89-5.00) | 0.09 |
| 4Q | 0.38 (0.07-1.90) | 0.24 | 0.36 (0.68-1.85) | 0.22 | 0.83 (0.32-2.15) | 0.71 | 0.97 (0.35-2.70) | 0.95 |
| J-index | 1.11 (0.96-1.28) | 0.17 | 1.13 (0.97-1.32) | 0.13 | 1.04 (0.92-1.17) | 0.50 | 1.05 (0.92-1.21) | 0.45 |
| 1Q | Ref |  | Ref |  | Ref |  | Ref |  |
| 2Q | 1.00 (0.14-7.21) | 1.00 | 0.97 (0.13-7.12) | 0.97 | 1.96 (0.75-5.09) | 0.17 | 2.27 (0.81-6.37) | 0.12 |
| 3Q | 5.35 (1.15-24.97) | 0.03 | 4.82 (0.99-23.4) | 0.05 | 3.41 (1.39-8.35) | 0.01 | 3.86 (1.44-10.3) | 0.01 |
| 4Q | 4.21 (0.88-20.24) | 0.07 | 4.49 (0.87-23.3) | 0.07 | 3.41 (1.39-8.35) | 0.01 | 3.69 (1.35-10.1) | 0.01 |

Adjusted variables; age, male, NIHSS score, TOAST classification, IAT, AF, previous stroke, SBP, RAO, and recanalization status.

Supplemental Figure S1. Serial glucose levels plotted according to the 3-month outcomes; (A) SICH vs. no SICH and (B) mRS 6 vs. mRS 0-5.


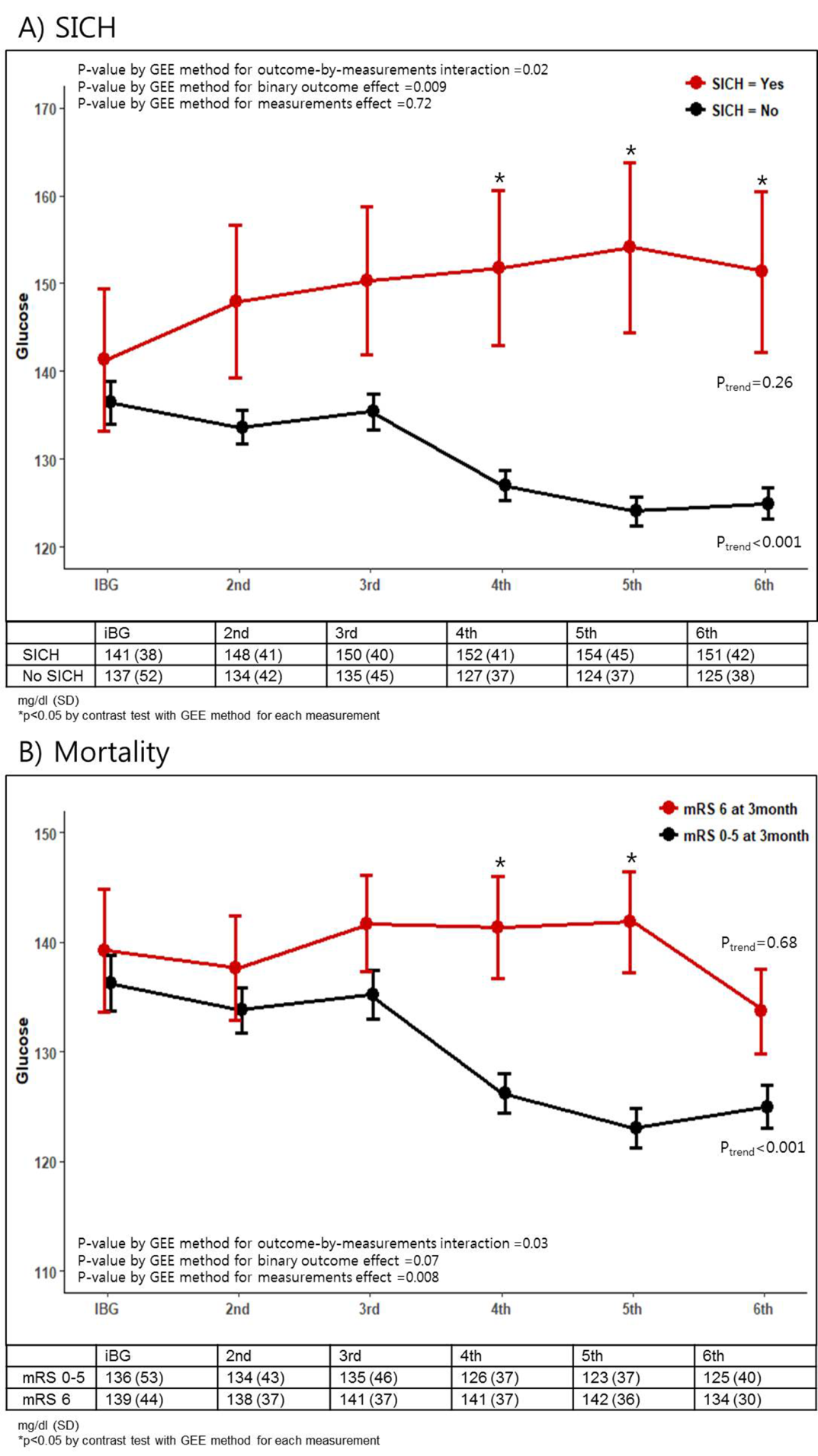

Supplement: Supplementary file 1 — Supplementary Information [file 41598_2018_30028_MOESM1_ESM.docx]
